# Lipid Metabolism Crosstalk in the Brain: Glia and Neurons

**DOI:** 10.3389/fncel.2019.00212

**Published:** 2019-05-21

**Authors:** Casey N. Barber, Daniel M. Raben

**Affiliations:** Department of Biological Chemistry, The Johns Hopkins University School of Medicine, Baltimore, MD, United States

**Keywords:** neurons, lipids, glial, CNS, communication

## Abstract

Until recently, glial cells have been considered mainly support cells for neurons in the mammalian brain. However, many studies have unveiled a variety of glial functions including electrolyte homeostasis, inflammation, synapse formation, metabolism, and the regulation of neurotransmission. The importance of these functions illuminates significant crosstalk between glial and neuronal cells. Importantly, it is known that astrocytes secrete signals that can modulate both presynaptic and postsynaptic function. It is also known that the lipid compositions of the pre- and post-synaptic membranes of neurons greatly impact functions such as vesicle fusion and receptor mobility. These data suggest an essential lipid-mediated communication between glial cells and neurons. Little is known, however, about how the lipid metabolism of both cell types may interact. In this review, we discuss neuronal and glial lipid metabolism and suggest how they might interact to impact neurotransmission.

Research on glial cells began in 1846, when Rudolf Virchow described a “substance” in which the various parts of the nervous system were embedded. In subsequent years, different kinds of glial cells were discovered and classified. Even though Santiago Ramon y Cajal was the first to propose a wide and diverse list of the functions of glia, these cells were viewed as simple neuronal support cells (García-Marín et al., 2007). In the last 20 years, however, studies have illuminated many functions of glia, highlighting their significance within the nervous system. In addition to providing many homeostatic functions to neurons, glial cells play a large role in regulating neurotransmission. Currently, much is known about lipid metabolism within neurons and glia separately, but little is known about potential lipid metabolic crosstalk between the two cell types. Here, we propose that lipid metabolism specifically from glial cells affects neurotransmission by regulating the rates of synaptic vesicle exo- and endocytosis. We will describe the known functions of glia and their lipid metabolism and then describe how glial lipid metabolism can possibly interact with neuronal lipid metabolism to regulate neurotransmission within the mammalian central nervous system.

Glial cells are the predominant cell type in the mammalian brain. They can contribute to 33–66% of the brain's total mass. Glial cells are segregated into three main classifications: astrocytes, microglia, and oligodendrocytes. Importantly, each cell type performs critical functions that sustain the health and function of the surrounding neurons. Astrocytes have many functions, among them are regulating water transport and ion levels in neurons, maintaining the blood brain barrier, and clearing glutamate from synapses to prevent neurotoxicity (Jäkel and Dimou, 2017). Microglia act as neuroprotectors by engulfing debris and cells in the process of apoptosis. Microglia actively surveil and respond to the state of functional synapses, such that they can sense dysfunctional synapses and influence information circuitry by either contributing to the repair or removal of a cell (Graeber and Streit, 2010). In the developing brain, microglia actively phagocytose some synaptic material and have a critical role in synaptic pruning during maturation (Paolicelli et al., 2011). Oligodendrocytes are well-known as the myelin-producing glial cells that insulate axons to ensure rapid electrical conduction and also provide trophic support to neurons (Simons and Trajkovic, 2006). Together glial cells provide an abundance of support to neurons, and defects in glial cells are implicated in many neurological diseases such as Alzheimer's disease, amyotrophic lateral sclerosis, and herpes encephalitis (Tracey et al., 2018).

As mentioned above, much is known about the variety of functions performed by these non-neuronal cells within the mammalian brain. Specifically, there is a wealth of information regarding the brain's energy utilization and how glial cells and neurons work together to support the brain's energy demands (Pellerin et al., 2007; Bélanger et al., 2011; Bruce et al., 2017; Tracey et al., 2018). This is crucial, as 20% of the body's total ATP consumption occurs in the brain, primarily for restoring ion gradients and motor-driven transport, as well as other functions (Bélanger et al., 2011). However, not nearly as much is known about lipid metabolism within glial cells and its potential neuronal implications. Here, we would like to propose that there is significant lipid metabolic crosstalk between glia and neurons that could influence neuronal function and neurotransmission. This could be yet another example of how glial cells regulate synaptic transmission and brain function overall.

Lipid molecules are key components of the brain's complex structure and function, with lipids comprising around 50% of the brain's dry weight. The lipid composition of neuronal and glial cell membranes has been shown to affect cell function and neurotransmission (O'Brien and Sampson, 1965; Puchkov and Haucke, 2013). The brain is mainly comprised of long-chain polyunsaturated fatty acids (LC-PUFAs) such as eicosapentaenoic acid, docosahexaenoic acid, and arachidonic acid. Importantly, several recent studies provide evidence that fatty acids, including essential fatty acids which must be obtained from the diet, can cross the blood-brain barrier (BBB) and be taken up by neurons via fatty acid transporters. Both neurons and astrocytes express several fatty acid transporters such as FATP1, FATP4, and CD36. It was recently shown that fatty acid binding protein 7 (FABP7) knockout mice exhibit schizophrenic phenotypes, such as deficits in prepulse inhibition, related to mutated spine morphology. Neurons from these mice had a reduction in dendritic complexity, spine density, and maturity of spines of pyramidal neurons (Watanabe et al., 2007; Ebrahimi et al., 2016; Bruce et al., 2017).

It is also widely thought that neurons receive metabolic support from astrocytes in a number of ways, one of which being that fatty acid oxidation primarily occurs in astrocytes. After which, the metabolites (ketones, NADH, acetyl CoA, FADH_2_) are taken up and utilized by neurons. Neurons are not energy-storing cells and do not normally contain a significant pool of lipid droplets or glycogen. Therefore, the available energy is depleted rapidly. This is one factor that makes them especially sensitive to stressful conditions such as continued stimulation. This is because the consumption of glycogen or fatty acids from lipid droplets is used in many other cell types as energy reserves. Periods of such high stimulation lead to increased reactive oxygen species (ROS) levels, which causes a buildup of peroxidated fatty acids. This is extremely harmful to neurons and must be prevented by removing or destroying the peroxidated fatty acids. In contrast to neurons, astrocytes store energy in the form of lipid droplets and act as a buffer to periods of toxic stimulation to neurons. Studies have shown that periods of high stress in neurons induce the formation of lipid droplets in nearby astrocytes, implying that overstimulated neurons may shuttle their peroxidated fatty acids to neighboring astrocytes (Bailey et al., 2015; Liu et al., 2015). This effect was found to be dependent on lipoproteins, the proteins responsible for trafficking fatty acids. In support of these studies, Ioannou et al. (2018) found that stressed neurons release peroxidated fatty acids bound to lipoproteins that are secreted by astrocytes or neurons. Neighboring astrocytes endocytose these lipoprotein-fatty acid particles and store the fatty acids in their lipid droplets. Astrocytes also react to overstimulated neurons by breaking down their lipid droplets and shuttling them to their own mitochondria for oxidative phosphorylation. Taken together, these data support the notion that astrocytes provide defense for overstimulated neurons by preventing fatty acid toxicity (Ioannou et al., 2018).

In addition to the metabolism of peroxidated lipids by astrocytes, most of the research regarding astrocytic lipid metabolism has been conducted specifically on cholesterol, and further research is required to study the metabolism of other lipids within astrocytes. Astrocytes and neurons both make cholesterol *de novo* but differ greatly in the pathways of cholesterol homeostasis and trafficking. Currently cholesterol metabolism within astrocytes serves as a model to illustrate the differences within neurons and astrocytes in pathways of synthesis, utilization, and efflux. Chen et al. (2013) found that astrocytes expel cholesterol with both lipid-free apolipoproteins and lipoproteins, while cholesterol efflux from neurons is induced only by lipoproteins. Lipoproteins are synthesized specifically by astrocytes and not neurons. While these proteins take cholesterol from neurons, they are also able to bind lipoprotein receptors within neuronal membranes that induce synaptogenesis (Chen et al., 2013).

Cholesterol is particularly enriched in synaptic membranes and affects a number of properties including endo- and exocytosis, lipid raft formation, and membrane fluidity, all of which greatly regulate neurotransmission. Therefore, the synthesis and transport of cholesterol is a modulating factor of synaptic signaling. Camargo et al. (2012) found that the synthesis of cholesterol, as well as fatty acids, within astrocytes is dependent on sterol regulatory element binding proteins (SREBPs). It was later found that components of the SREBP pathway are most highly expressed in hippocampal astrocytes. Importantly, a decrease in SREBP activity in astrocytes leads to a defect in synaptic function and plasticity. Mutated proteins within the astrocyte SREBP pathway caused a defect in synaptic structure, vesicle populations, and presynaptic function. Specifically, synapses in mice in which the SREBP cleavage-activating protein (SCAP) was deleted from GFAP-expressing cells were found to have lower levels of SNAP-25, a crucial SNARE protein involved in vesicle fusion with the presynaptic membrane (van Deijk et al., 2017). This is a critical example of how elements produced within glial cells can be transferred to neurons to affect synaptic transmission.

The phospholipid composition of presynaptic membranes contributes to the regulation of the rates of synaptic vesicle exo- and endocytosis. The shapes of particular lipids affect the membrane curvature and make the architecture more or less susceptible to vesicle fusion. Cholesterol, diacylglycerol, and phosphatidic acid are cone-shaped lipids and induce negative membrane curvature, promoting membrane fusion (Ammar et al., 2013). In addition to affecting membrane curvature, these lipids also bind important protein regulators of vesicle fusion, such as syntaxin-1A, NSF, and small GTPases (Jang et al., 2012). Therefore, portions of the membrane that are more highly enriched in these cone-shaped lipids are potentially more likely to undergo vesicle fusion, highlighting the phospholipid composition of synaptic membranes as a regulating factor of synaptic vesicle cycling.

So how is the lipid composition of membranes determined? What factors play a role in regulating the relative amounts of lipids within membranes? One of the most important factors are lipid-metabolizing enzymes, such as diacylglycerol kinases (DGKs) and phospholipases, that catalyze the conversion of these lipids. For example, DGKs and phospholipases D (PLDs) both produce phosphatidic acid from different substrates (Puchkov and Haucke, 2013). As phosphatidic acid is highly implicated in vesicle fusion, these enzymes are thought to be regulators of the synaptic vesicle cycle. In 2016, Goldschmidt et al. found that knockout of a particular DGK, DGKθ, in neurons resulted in significantly decreased rates of endocytosis after stimulation, in comparison to wildtype neurons. This is just one study, in addition to many others, that has discovered proteins with a regulatory role in the synaptic vesicle cycle (Rosahl et al., 1995; Südhof and Rizo, 1996; Wang et al., 1997; Turner et al., 1999; Reim et al., 2001; Pechstein et al., 2010; Lai et al., 2017). Many of these characterized proteins can be categorized by their mechanism of action on the SV cycle: regulation of calcium influx, regulation of SNARE dynamics, changes in membrane curvature, or synaptic vesicle priming. However, in some cases, the exact molecular actions of regulatory proteins have not been discovered. Being that the lipid membrane is the physical barrier between an intact synaptic vesicle and the synaptic cleft, it is not hard to imagine that the last regulatory step of vesicle fusion could be the lipid composition of the presynaptic membrane. This being so, the hypothesis that many SV regulatory proteins are lipid-metabolizing proteins is not inconceivable.

In addition to proteins, other factors influence the phospholipid composition of membranes. As discussed above, astrocytes synthesize lipids, and traffic them to neurons, offering another point of regulation by astrocytes. Also, they secrete signals that can influence synaptic transmission. These are often signaling factors that have downstream effects that influence synaptic vesicle fusion (Jäkel and Dimou, 2017). What about astrocytic factors that have a more direct impact on the synaptic vesicle cycle? We hypothesize that astrocytes have a direct role in regulating synaptic transmission via astrocyte lipid metabolism followed by transport to neurons. We suggest that astrocytes might be responsible for making some of the lipids that are directly inserted into the presynaptic plasma membrane. Other proteins, such as flippases, are likely involved that flip lipids into the inner membrane leaflet and sort these lipids into microdomains that make certain areas of the membrane susceptible for fusing with synaptic vesicles (Andersen et al., 2016). Astrocytes are involved in the transportation of several molecules and proteins to neurons, so membrane lipids such as phosphatidic acid could feasibly be transferred directly or indirectly from astrocytes to neurons. In fact, Tabernero et al. (2001) found that astrocytes secrete oleic acid and suggested that this is taken up by neurons for the process of phospholipid synthesis during neuronal differentiation. In addition to lipids, studies have also found that astrocytes secrete or release: large vesicles containing functional mitochondria, ATP, and lipid droplets (Falchi et al., 2013); dense-core vesicles containing neuropeptide Y (Ramamoorthy and Whim, 2008); glucose, lactate, glutamine, and glutamate through gap junctions connecting astrocytes (Giaume et al., 1997; Gandhi et al., 2009); thrombospondins and hevin, which act on neurons to promote synaptogenesis (Christopherson et al., 2005; Kucukdereli et al., 2011); and many other molecules and proteins such as glypicans, Wnts, and tumor necrosis factor α (TNFα) (Allen, 2014). Therefore, there is much evidence that astrocytes release many factors that affect neurons, and we propose that lipids are also an important astrocyte-secreted factor.

Astrocytic processes are intimately involved with synapses, comprising the tripartite synapse. Astrocytes have a wide, radial branching morphology, allowing them to physically protrude into the synaptic cleft between pre- and postsynaptic boutons and exchange information with these elements, contributing to synaptic physiology (Perea et al., 2009). Therefore, it is conceivable that lipids may be transferred within the synaptic region of the neuron because of how closely related neurons and astrocytes are in physical space. In support of this hypothesis, Zhu et al. (2016) found that phosphatidic acid produced specifically from astrocytes regulates neurite outgrowth and dendritic branching in neurons. This study found that knockdown of PLD1 only in astrocytes led to a reduction in secretion of phosphatidic acid and a decrease in dendritic branching of neurons. Furthermore, neurons grown in the presence of conditioned media from astrocytes with PLD1 knockdown had significantly decreased dendritic branching. This study is an example of how secreted lipids from astrocytes can affect functional aspects of neurons.

Astrocytes could also synthesize other proteins that have regulatory roles in the synaptic vesicle cycle, thereby having a more indirect effect on neurotransmission. For instance, it is possible that specific lipid-metabolizing enzymes could be expressed only within glial cells and that mechanisms transfer the products of the reactions they catalyze from glial cells to neurons to affect membrane architecture. We hypothesize that PLD may be implicated in synaptic vesicle exocytosis. The localization and expression of PLD is currently unclear (Saito et al., 2000; Ammar et al., 2015; Vermeren et al., 2016), although some studies suggest that PLDs are localized within glial cells (Jin et al., 2002; Zhang et al., 2004; Zhu et al., 2016). The localization and expression pattern of PLDs in the brain needs to be clarified, but it is possible that PLDs are selectively expressed in astrocytes. If this is the case, astrocytes could produce phosphatidic acid by PLD that is trafficked to or taken up by neurons to influence the dynamics of synaptic vesicle fusion (Figure 1).

**Figure 1:**
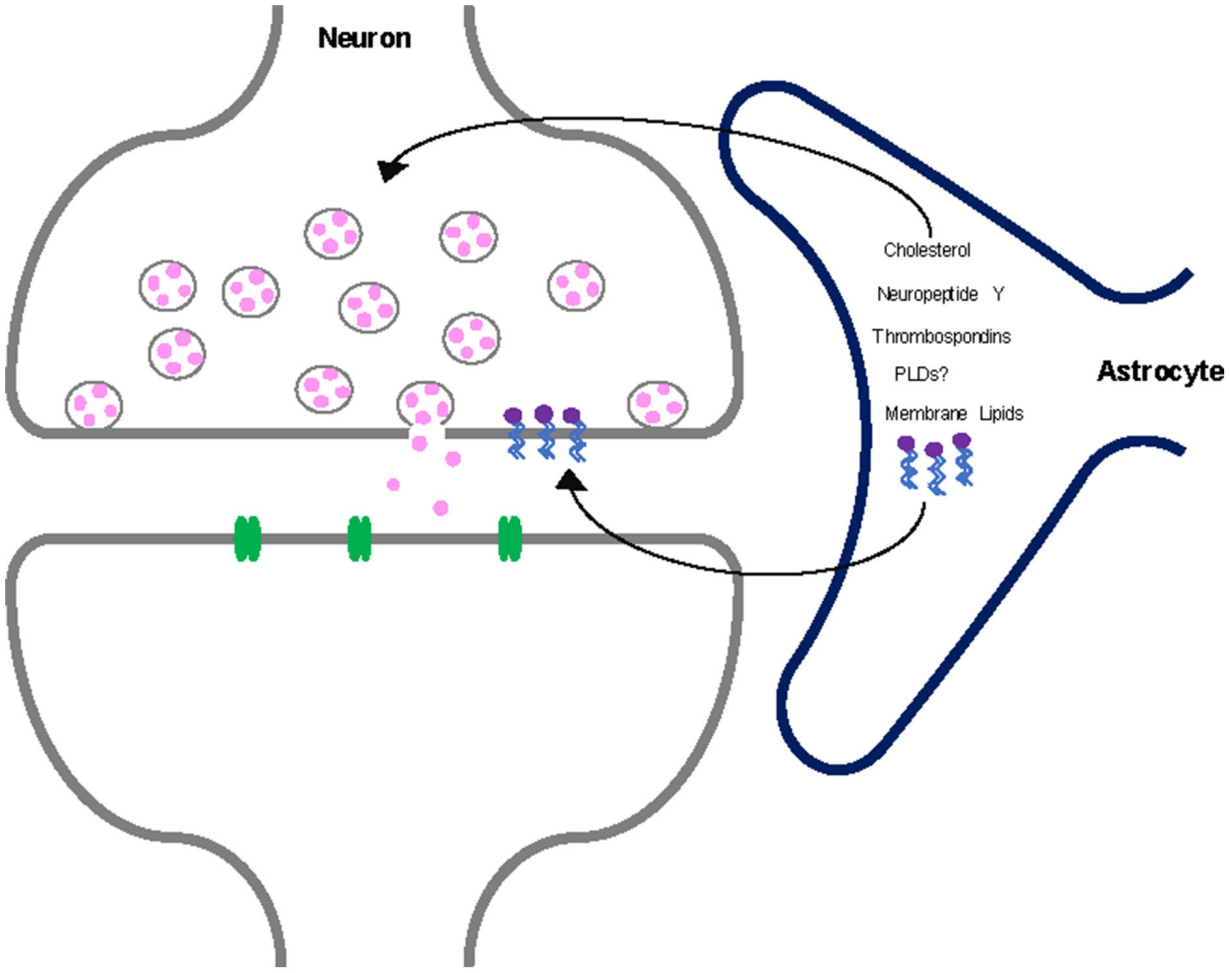
Astrocytes secrete many factors that are critical to neuron function such as cholesterol, neuropeptide Y, thrombospondins, and other metabolic intermediates. We hypothesize that membrane lipids are also secreted by astrocytes and taken up by neurons to regulate neurotransmission. Additionally, PLDs selectively expressed within astrocytes may contribute to this astrocytic lipid metabolism.

In conclusion, we believe that glial cells have a specific role in regulating synaptic transmission in neurons via lipid metabolism. The relative amount of certain lipids within neuronal membranes impacts the efficiency of endo- and exocytosis, and this could be developed by lipid synthesis or metabolism within glial cells. Further research is required to determine the expression of lipid-metabolizing enzymes within glial cells and pathways of lipid metabolism and trafficking. Regardless, astrocytes, microglia, and oligodendrocytes are vital components of the nervous system that contribute to regulating many neuronal functions, including synaptic transmission.

## Author Contributions

All authors listed have made a substantial, direct and intellectual contribution to the work, and approved it for publication.

### Conflict of Interest Statement

The authors declare that the research was conducted in the absence of any commercial or financial relationships that could be construed as a potential conflict of interest.
